# Oral Health and Molecular Aspects of Malignant Fibrous Histiocytoma Patients: A Systematic Review of the Literature

**DOI:** 10.3390/ijerph17041426

**Published:** 2020-02-23

**Authors:** Khrystyna Zhurakivska, Giuseppe Troiano, Marco Montella, Lorenzo Lo Muzio, Luca Fiorillo, Gabriele Cervino, Marco Cicciù, Cesare D’Amico, Rosario Rullo, Gregorio Laino, Dario Di Stasio, Luigi Laino

**Affiliations:** 1 Department of Clinical and Experimental Medicine, University of Foggia, 71122 Foggia, Italy; khrystyna.zhurakivska@gmail.com (K.Z.); giuseppe.troiano@unifg.it (G.T.); lorenzo.lomuzio@unifg.it (L.L.M.); 2 Multidisciplinary Department of Medical-Surgical and Dental Specialties, Second University of Naples, 80100 Naples, Italy; montella.marco19@gmail.com (M.M.); rosario.rullo@unicampania.it (R.R.); gregorio.laino@unicampania.it (G.L.); dario.distasio@unicampania.it (D.D.S.); luigi.laino@unicampania.it (L.L.); 3 Department of Biomedical and Dental Sciences, Morphological and Functional Images, University of Messina, Policlinico G. Martino, Via Consolare Valeria, 98100 Messina ME, Italy; gcervino@unime.it (G.C.); mcicciu@unime.it (M.C.); cdamico@unime.it (C.D.)

**Keywords:** MFH, UHGPS, oral histiocytoma, oral sarcoma, oral medicine, oral pathology, oral health, prevention, mouth

## Abstract

Malignant fibrous histiocytoma is one of the most common soft tissue sarcomas in adults. It occurs only occasionally in oral soft tissues, and knowledge about its characteristics is based on a limited number of cases reported in the literature. Malignant fibrous histiocytoma belongs to the group of soft tissue sarcomas and makes up less than 10% of soft tissue sarcomas. For therapeutic purposes, complete exeresis of the lesion (macroscopic and microscopic) is performed because they have frequent recurrences. As for complementary therapy in addition to surgery, neither radiotherapy nor chemotherapy have been shown to reduce the risk of death related to the disease. Often patients complain of a swelling that grows in a short period of time. It is quite common for patients to report trauma in the area, which is not the cause, but rather the event that allows diagnosis. The mass usually does not cause pain unless it compresses an adjacent nerve structure. The aim of this study is to systematically review the scientific literature in order to identify the most recent studies concerning malignant fibrous histiocytomas localized in oral soft tissues and report their main data. The main outcomes of this study concern the immunohistochemical, molecular, and clinical aspects of this pathology. A systematic review of articles in the electronic databases pubmed, Scopus, and Web of Science was performed. After the selection process, 11 studies met the inclusion criteria and were included in the review. The mean age of the patients was 50.8 years old. The lesions affected various parts of the oral cavity, showing predominantly storiform–pleomorphic patterns. All cases except one were treated with surgical resection and radiation therapy. Although some data emerged from this review, they remain limited to a few case reports. Further studies are necessary in order to standardize the approach to patients affected by oral malignant fibrous histiocytoma (MFH).

## 1. Introduction

### 1.1. Rationale

Malignant fibrous histiocytoma (MFH) is an aggressive primitive histological variant of high-grade sarcomas [1,2]. It was described as a distinct clinicopathologic entity for the first time by O’Brien and Stout in 1964 [3] and considered to be one of the most common soft tissue sarcomas in adults [4].

Today, it is estimated to be the second most common soft tissue sarcoma, with an incidence of 0.88 cases per 100,000 annually, and a male:female ratio of 2:1, appearing predominantly during the 6th and 7th decades of life [5].

The first microscopical characterization described a storiform growth pattern of fibroblasts that acquired phagocytic properties. A histiocytic origin has been suggested based on the morphology of the cell pattern. In 1978, a large cohort study on the MFH was conducted; the results identified different morphologic phenotypes, among which the storiform–pleomorphic appeared the most prevalent, followed by myxoid, giant-cell, inflammatory, and angiomatoid subtypes [6].

In 2002, the World Health Organization (WHO) reclassified MFH under the term of undifferentiated high-grade pleomorphic sarcoma (UHGPS) [7,8]. However, even after the new classification, the MFH term is still widely used. In 2014, Delisca et al. [9] conducted a retrospective study in order to determine the presence of clinical prognostic implications that have evolved with this new nomenclature. They compared outcomes of patients diagnosed with MFH with those diagnosed with UHGPS and concluded that no identifiable prognostic implications seem to exist.

The head and neck region are rarely affected, compared with other regions. In fact, the head and neck involvement is estimated to be only 3–7%, compared with almost 50% of tumors that occur on an extremity or in the abdominal cavity and retroperitoneum [4,10]. In particular, the soft tissues of the craniofacial region are only occasionally affected by MFH; the sinonasal tract (30%) and the facial skeleton (15–25%) are the most common head and neck sites [11]. Several cases of MFH affecting mandible, maxilla and maxillary sinus have been also reported in literature [12,13,14,15]. Concerning the soft tissues of the oral cavity, knowledge about MFH/UHGPS is limited to a small number of cases described in the literature [1,16,17,18]. The most common clinical presentation consists of a painless exophytic mass that may or not be ulcerated [19]. Distant metastases of MFH commonly occur via hematogenous or lymphatic spread [1,6,20,21]. Unfortunately, it is not always possible to intervene in a timely manner in these cases, due to delays related to diagnosis or systemic conditions of the patient who need adequate protocols or therapies [22,23,24]. Many syndromic patients or those suffering from systemic diseases may also present intra and post-operative complications [23,25,26] or represent a high post-surgical infection risk [27,28,29] and therefore need adequate therapies [27,30].

### 1.2. Objectives

The aim of the present review is to systematically analyze the recent scientific literature in order to identify the reported cases of MFH/UHGPS localized in oral soft tissues, collecting data about the age and the gender of patients, localization and size of the lesion, immunohistochemical evaluation, the treatment choice, and the follow-up period with eventual recurrence.

## 2. Materials and Methods

### 2.1. Protocol and Registration

This systematic review was performed according to the Preferred Reporting Items for Systematic reviews and Meta-Analysis (PRISMA).

### 2.2. Eligibility Criteria

The criteria for inclusion in this systematic review were:

- Original studies or case reports that presented diagnoses of MFH/UHGPS with primary localization in the oral cavity soft tissues.

Exclusion criteria were:

- Studies that reported radiation-induced MFH;
- Studies that reported oral metastasis of MFH originating in other regions;
- Studies written in other languages than English;
- Systematic reviews that reported cases of MFH/UHGPS without present patient data and/or features of the lesions.

**Figure 1:**
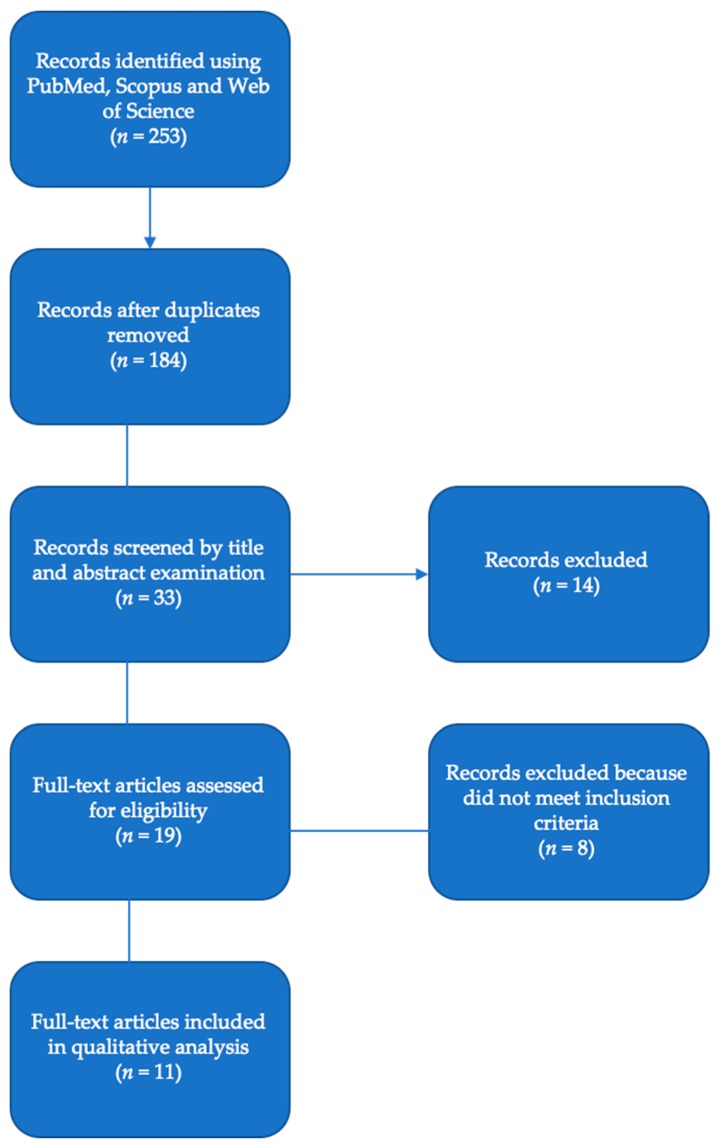
Flow chart according to PRISMA statement.

Research results that did not meet the inclusion criteria were excluded during the data collection process (Figure 1).

### 2.3. Information Sources

A systematic review of English articles in the electronic databases pubmed, Scopus, and Web of Science was conducted independently by two authors (K.Z. and G.T.) using search terms.

### 2.4. Search

“Malignant fibrous histiocytoma” AND (oral OR tongue OR lips OR cheeks). An additional search in the aforementioned databases was conducted using the new classification nomenclature “High-Grade undifferentiated pleomorphic sarcoma” AND (oral OR tongue OR lips OR cheeks).

### 2.5. Data Collection Process

No restrictions were imposed concerning study design; searches included articles published between 1997 and 2017.

Furthermore, the bibliography of the included articles was examined in order to find other studies to include in the review.

A first selection was performed reading the titles and the abstracts of the search results. After this round, duplicates from the different databases were removed. The studies that appeared eligible were included for full-text reading. Once the full-text evaluation was completed, only studies meeting all inclusion criteria and considered eligible by both authors were included in the review. Disagreements between the authors were resolved through discussion.

### 2.6. Data Items

As shown in Table 1, considered data items are:

- Year;
- Number of patients;
- Age;
- Sex;
- Localization;
- Size;
- Immunohistochemical evaluation (+);
- Histotype Lymph node involvement;
- Treatment;
- Follow-up;
- Recurrence.

### 2.7. Risk of Bias in Individual Studies

Since the included studies are single patient case reports, it was not possible to estimate an effect size or quality assessment [31]. Consequently, only descriptive results were collected and reported.

## 3. Results

### 3.1. Study Selection

A total of 253 titles and articles were screened in the first round of the selection process. Thirty-three studies were identified as acceptable for full-text evaluation, and after duplicates were removed, 19 articles were read in full. By the end of this stage, only 11 studies [19,32,33,34,35,36,37,38,39,40,41,42,43] met inclusion criteria and were included in the qualitative analysis. Eight were excluded [10,44,45,46,47,48,49,50] because they did not comply with inclusion criteria. The flowchart in Figure 1 represents the selection process for study inclusion.

### 3.2. Study Characteristics

For each study, the following data were extracted: number of patients, age, sex, localization and size of the lesion, histologic and immunohistolochemical evaluation if reported, the lymph node involvement if present, the treatment choice, the follow-up period, and eventual relapse of the disease.

### 3.3. Results of Individual Studies

Of the 11 included studies, all were case reports, presenting 1 case for each study. The 11 considered patients were 5 females and 6 males, with a mean age of 50.8 years, and range between 20 and 82 years. Regarding the site of localization, gums were involved in 4 cases [32,33,36,40]; the tongue was affected in 3 patients, of which 2 cases concerned the lateral surface [19,41]; and the base of the tongue in one case [39]. In one case, the lesion manifested on the oral mucosa [34,35]; another case involved the palate [38] and one the floor of the mouth [43]. The dimensions of the lesions were reported in 10 cases, and the average diameter was approximately 3 cm. Regarding histotype, 6 cases of histiocytoma presented a storiform–pleomorphic pattern [32,33,40,41,43], one study reported undifferentiated high-grade pleomorphic sarcoma [38], and another one myxoid variant [34] of histiocytoma, while in 3 studies [35,36,39], no specification of histologic subtype was reported. Immunohistochemical positivity for vimentin was reported in 6 studies [32,34,35,38,39,43]; in 5 of these, it was associated with positivity for CD68 [32,34,38,39,43]. Positivity for reticulin was revealed in one study [36], while in another, anti-α_1_-chymothrypsin and α-smooth muscle actin positivity [19] were detected. Only one study reported lymph node involvement (Regarding treatment, surgical resection was performed in all studies except one, in which the type of therapy was not reported [33]. Three patients were subjected to partial maxillary bone resection [36,38,40], while three other studies reported association of the surgery with adjuvant radiation therapy). Only 7 of the included studies presented a follow-up period; this was of 17.1 months on average, with a minimum of 3 [36] and a maximum of 28 months [41]. Only one study reported a relapse of the disease 2 weeks after a first surgery consisting of local excision with maxillectomy [38], while 4 studies made no reference to recurrence [32,33,34,40]. These invasive surgeries often require post-surgical rehabilitations [51,52], and in some cases are made complex by the presence of local contraindications (noble anatomical structures [53,54,55,56], or systemic contraindications (general health conditions of the patient [22,23,24,26,57]). Post-surgical and infection management are important [27,28,30].

## 4. Discussion

### 4.1. Summary of Evidence

Malignant fibrous histiocytoma is one of the most common soft tissue sarcomas in adults. Its occurrence in oral soft tissues is rare, and only single cases are reported in the literature. Consequently, diagnostics and therapeutic approaches lack substantial scientific support, posing various difficulties for clinicians. The peak of incidence is estimated to be in the seventh decade, affecting predominantly males [6]. Notably, the results of our analysis revealed that in oral cavity soft tissues, MFH seems to occur also in younger patients, showing a mean age of about 50 years, including one patient who was 42 years old. Histological diagnosis of MFH can be difficult, especially when biopsy specimens are not dimensionally sufficient; multiple biopsies are sometimes required. Despite the heterogeneity of the lesions, MFH has a fibroblastic nature and shows a facultative histiocytic differentiation. The pleomorphic–storiform subtype is the most common and is characterized by plumper fibroblast-like cells with evident nuclear atypia, a great number of histiocyte-like round cells, and pleomorphic giant cells.

Due to its pleomorphic spindle cell morphology, differential diagnosis using immunohistochemical evaluation (IHC) should be performed. Immunohistochemical analysis usually demonstrates a “vimentin-only” phenotype, excluding some other neoplasms such as sarcomatoid variants of squamous cell carcinoma, spindle cell carcinoma, vascular tumors, or synovial sarcoma—showing negative to their markers.

Considering the reclassification developed by WHO in 2002, MFH was renamed as “undifferentiated high-grade pleomorphic sarcoma” (UHGPS) in order to better clarify the origin of tumor cells. In 2012 WHO clarified that diagnosis of UHGPS could be made only if “no definable line of differentiation” could be established. Despite the new classification, the previous term MFH was maintained and many clinicians and pathologists continue to use it in their practice.

In order to include all studies recorded both with the old and the new nomenclatures, two searches were carried out on all databases: using the old nomenclature, terming “malignant fibrous histiocytoma”, as well as the new terms “high-grade undifferentiated pleomorphic sarcoma”. Only one of the included studies was identified by the second search using the new nomenclature. [43] However, as demonstrated by Delisca et al. [9], no difference seems to be present in outcomes between patients designated historically in the MFH group and those included in the new UHGPS classification.

All regions of the mouth could be affected and, in the cases analyzed, the mean dimension of the neoplasm was about 3 cm at the moment of diagnosis. For large lesions, incisional biopsies were initially performed, followed by a surgical excision or, in some cases, by a wide resection.

Regarding prognosis in head and neck sarcomas, tumor size and tumor grade seem to have greater prognostic importance than margin status [58]. In particular, the data regarding the prognosis of MFH/UHGPS suggest a 19–31% recurrence rate, 31–35% metastasis rate, and a 5-year survival rate of 65–70%. Generally, prognosis of sarcomas involving intraoral sites is considered to be better as compared to extraoral sites, [59] but data related to MFH are poor. In the present review, only one study reported the recurrence of the neoplasm [38] that occurred 2 weeks after the first resection. It was a big lesion, involving the alveolar process, body of the maxilla, and side of the hard palate. Only in this case, multiple enlargement of lymph nodes in bilateral submandibular and upper deep cervical regions were also noted. The surgical treatment of these lesions unfortunately has intraoperative difficulties that may be related to the systemic conditions of our patients, or to local contraindications, represented by the presence of noble anatomical structures or intraoperative difficulties due to this [54,55,56,60,61,62,63]. Surgical resection is the mainstay of treatment for MFH of the maxillary sinus. A radical resection with clear margins is essential for excellent local control and long-term survival [64]. Head and neck malignant fibrous histiocytoma present at a smaller size and lower grade, likely due to earlier presentation in this region. Because of this, head and neck malignant fibrous histiocytoma represents a favorable survival prognosis compared with extremity disease [65,66].

In the other included studies, no relapse was reported, but data on long-term follow-up are lacking, providing a maximum of 28 months of follow-up in one considered case.

### 4.2. Limitations

The present review presents several limitations due to the quality of the included studies. Since the object is represented by a rare condition, the literature lacks randomized studies. However, case reports are important for the initial detection and description of rare diseases.

### 4.3. Conclusions

Recent data in the literature concerning oral MFH/UHGPS are still limited to individual experiences presented with case reports. Data on treatment, follow-up, and survival are also limited and insufficient for guidance in approaching patients with MFH. Furthermore, there is a confusion related to the nomenclature and histological diagnosis of the pathology, since the new classification has not yet been adopted by many pathologists. Further studies and attention to approaches to MFH localized in the oral cavity are necessary to ensure a scientific and evidence-based approach.

## Figures and Tables

**Table 1:** Data for: name of the study, year of publication, number of the patients, age of the patient, sex of the patient, localization and dimension of the lesion, immunohistochemical positivity, histotype, lymph node involvement, treatment, follow-up period, and eventual recurrence.

Authors	Year	Number of Patients	Age	Sex	Localization	Size	Immunohistochemical Evaluation	Histotype	Lymph Node Involvemen	Treatment	Follow-Up	Recurrence
Vijayalakshmi et al. [32]	2012	1	60	F	Gingiva (upper maxilla)	4 × 3 cm	vimentin, CD 68	Storiform–pleomorphic	No	excisional biopsy	----	----
Park et al. [33]	2009	1	41	F	Gingiva (upper maxilla)	2.5 cm	------	Storiform–pleomorphic	-----	-----	------	-----
Balaji [34]	2010	1	31	F	buccal mucosa	2.7 × 2.5 × 2.5 cm	vimentin, CD 34, CD68. Isolated areas of S100 positivity	Myxoid variant	No	surgical resection	----	-------
Rapidis et al. [19]	2005	1	24	M	side of the tongue	3 × 2 × 1 cm	anti-α_1_-chymothrypsin, α-smooth muscle actin, CD-68	Storiform–pleomorphic	No	local resection with 1-cm tumor-free margins	18 months	no
Nagano et al. [35]	2008	1	61	M	Buccal mucosa	2.5 × 5.0 cm	vimentin	-----	No	excision + external radiotherapy + CyberKnife therapy	22 months	no
Agnihotri et al. [36]	2008	1	20	F	Gingiva (Upper maxilla)	2 × 2.5 cm	reticulin	-----	No	resection of the premaxilla	3 months	no
Boaz et al. [38]	2017	1	55	F	Gingiva (palate)	8.2 × 5.5 × 4.5 cm	Vimentin, focally positive for CD 68	Undifferentiated High-Grade Pleomorphic Sarcoma	Yes	maxillectomy	12 months	Yes after 2 weeks
Dhingra et al. [39]	2012	1	57	M	Base of tongue	3 × 2 × 2 cm	CD68 and vimentin	-----	No	surgical excision with 1-cm healthy margins	12 months	no
Bali et al. [40]	2010	1	72	M	Gingiva (upper maxilla) and labial mucosa with invasion of the underlying bone	----	------	Storiform–pleomorphic pattern	no	wide surgical excision of the soft tissue mass with a segment of the maxillary bone	-----	-------
Canyilmaz et al. [41]	2001	1	82	M	lateral surface of the tongue	3 × 3 cm	--------	Storiform–pleomorphic	no	tumoral excision with wide resection margins + adjuvant radiotherapy	28 months	no
Alfredo et al. [43]	2007	1	56	M	floor of the mouth	4.5 cm	Vimentin, CD 68	Storiform–pleomorphic	no	surgical resection associated with adjuvant radiation therapy	25 months	no
